# High‐Density Artificial Synapse Array Consisting of Homogeneous Electrolyte‐Gated Transistors

**DOI:** 10.1002/advs.202305430

**Published:** 2023-11-28

**Authors:** Jun Li, Yuxing Lei, Zexin Wang, Hu Meng, Wenkui Zhang, Mengjiao Li, Qiuyun Tan, Zeyuan Li, Wei Guo, Shengkai Wen, Jianhua Zhang

**Affiliations:** ^1^ School of Material Science and Engineering Shanghai University Jiading Shanghai 201800 P. R. China; ^2^ Key Laboratory of Advanced Display and System Applications Ministry of Education Shanghai University Shanghai 200072 P. R. China; ^3^ School of Microelectronics Shanghai University Jiading Shanghai 201800 P. R. China; ^4^ Central Research Institute BOE Technology Group Company, Ltd. Beijing 100176 P. R. China

**Keywords:** artificial synapse array, electrolyte‐gated transistors, lateral‐gate, Photo‐Lithography, metal‐organic framework

## Abstract

The artificial synapse array with an electrolyte‐gated transistor (EGT) as an array unit presents considerable potential for neuromorphic computation. However, the integration of EGTs faces the drawback of the conflict between the polymer electrolytes and photo‐lithography. This study presents a scheme based on a lateral‐gate structure to realize high‐density integration of EGTs and proposes the integration of 100 × 100 EGTs into a 2.5 × 2.5 cm^2^ glass, with a unit density of up to 1600 devices cm^−2^. Furthermore, an electrolyte framework is developed to enhance the array performance, with ionic conductivity of up to 2.87 × 10^−3^ S cm^−1^ owing to the porosity of zeolitic imidazolate frameworks‐67. The artificial synapse array realizes image processing functions, and exhibits high performance and homogeneity. The handwriting recognition accuracy of a representative device reaches 92.80%, with the standard deviation of all the devices being limited to 9.69%. The integrated array and its high performance demonstrate the feasibility of the scheme and provide a solid reference for the integration of EGTs.

## Graphical abstract

A synaptic device array consisting of 100 × 100 electrolyte‐gated transistors are integrated into a TFT glass of 2.5 mm × 2.5 mm using photo‐lithography through a lateral‐gate structure. With competitive and homogeneous long‐term plasticity benefit from electrolyte framework of PEO/PVP/LiTFSI/ZIF‐67, the array performed complicated synaptic functions, providing a solid reference for the integration of EGT synaptic devices.

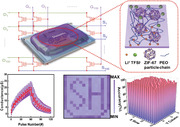

## 1 Introduction

Artificial synapse arrays can be employed to construct hardware neural networks, enabling the realization of neuromorphic computation with potential applications in intelligent perception, machine learning, and image processing.^[^ ^1^, ^2^, ^3^, ^4^, ^5^ ^]^ For example, professor Xu concentrates on environmental‐responsive artificial neuromuscular systems consisting of artificial synapse array to control artificial muscles according to external stimulus,^[^ ^6^, ^7^ ^]^ which is a favorable application of artificial synapse array. High‐density artificial synapse arrays can enhance the computational capacity and reduce the power consumption, thereby establishing more efficient artificial intelligence systems.^[^ ^8^, ^9^, ^10^ ^]^ However, current research is primarily focused on unit synaptic device, and the integration of high‐density synapse arrays with excellent performance and good homogeneity remains limited.^[^ ^11^, ^12^ ^]^ Among the synaptic devices, electrolyte‐gated transistors (EGTs) have gained considerable attention owing to their multiple terminals, easy coupling, and low power consumption.^[^ ^13^, ^14^, ^15^, ^16^ ^]^ Furthermore, an organic polymer electrolyte with low Young's modulus, which is beneficial for implantable and flexible electronics, can dissociate free ions and enable the working of EGTs at room temperature.^[^ ^17^, ^18^ ^]^ Consequently, EGTs based on organic polymer electrolyte are promising candidate as array units for future applications.

However, polymer electrolytes conflict with photo‐lithography, which is essential for implementing high‐density arrays with miniaturized and accurate pattern, since polymer electrolytes can be easily deteriorated by photoresist, and can lose their ionic activity.^[^ ^19^, ^20^ ^]^ This limits the realization of integrated arrays and the improvement of synaptic functions. Few studies have been conducted thus far on the integration of EGTs, and the existing studies implement simple simulations based on the characteristic index obtained from the synaptic functions of a single device, without considering that the performance of an artificial synapse array depends on the base of the real arrays.^[^ ^21^, ^22^, ^23^ ^]^ Although some studies have attempted to integrate real synaptic devices, this integration has been limited to a small scale and low density.^[^ ^24^, ^25^, ^26^ ^]^ For example, Roe et al. developed a 3 × 3 EGT array with a single synaptic device including a selective transistor and an EGT using a lateral gate structure, demonstrating the feasibility of the integration of EGTs.^[^ ^25^ ^]^ Some studies have focused on polymer processing with photo‐lithography to address the challenge faced by the integration process of EGTs that is caused by the incompatibility between organic polymer electrolytes and photo‐lithography.^[^ ^27^, ^28^, ^29^ ^]^ For instance, Gluschke et al. used electron–beam lithography to pattern Nafion, a commercial proton exchange membrane, causing it to achieve excellent protonic properties.^[^ ^28^ ^]^ However, a simple and universal method of integrating EGTs into an array with high density must be developed. Herein, we employed the lateral gate structures to delay the fabrication of the gate dielectric as the final step, which prevents the conflict between the polymer electrolyte and photo‐lithography. A trial‐and‐error test was rapidly and conveniently conducted on the synaptic transistors by changing the electrolyte, using the prepared array substrate and specific electrolytes.

Furthermore, we designed a composite electrolyte framework with high ionic conductivity, called poly(ethylene oxide) (PEO)/ polyvinyl pyrrolidone (PVP)/lithium bis(trifluoromethanesulphonyl)imide (LiTFSI)/zeolitic imidazolate framework‐67 (ZIF‐67), to enhance the array functions. Currently, ionic conductivity of polymers is improved by using soluble salts to increase the density of ions and inorganic solid‐state electrolytes with large specific areas to provide ionic pathways, thus increasing the ionic mobility.^[^ ^30^ ^]^ LiTFSI was selected as the salt owing to its effective ionic conductivity with heat, water, and electrochemical stability.^[^ ^31^, ^32^ ^]^ ZIF‐67, a cobalt‐based metal–organic framework (MOF) material, different from inorganic solid‐state electrolytes in comprising organic ligands and metal ions, simultaneously improves the porosity and maintains the zeolite stability,^[^ ^33^, ^34^ ^]^ therefore was selected as the ionic pathways provider. ZIF‐67 functions as an ionic pathway for the fast transport of ions in electrolytes, owing to its visible porous structures and hopping sites built on organic ligands.^[^ ^35^, ^36^, ^37^ ^]^ The metal ions can react with TFSI^−^ and weaken the coordination environment of Li^+^, rendering Li^+^ sufficiently active.^[^ ^35^, ^38^, ^39^ ^]^ Therefore, we have added LiTFSI and ZIF‐67 to significantly improve the ionic conductivity. Thus, the novel PEO/PVP/LiTFSI/ZIF‐67 composite is expected to achieve a quasi‐solid electrolyte with high ionic conductivity and stability for EGT arrays.

**Figure 1:**
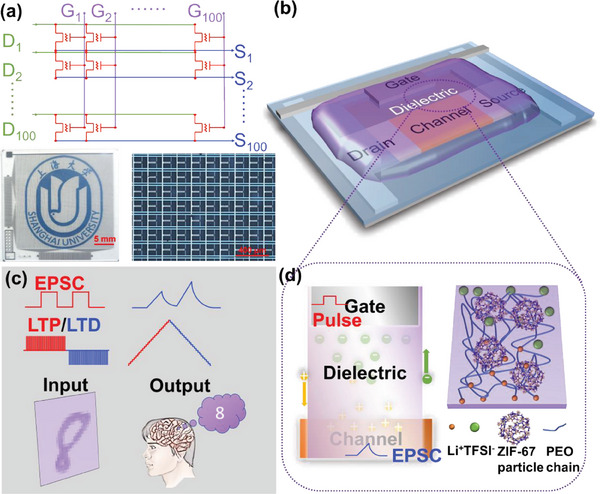
Structure of the electrolyte‐gated transistor (EGT) synaptic devices and the array. a) Diagram of the circuit, photograph, and micrograph of the array without electrolyte. 100 × 100 Transistors are integrated into a 2.5 × 2.5 cm^−^2 glass. Height of badge is 2.5 cm. b) Multi‐layer structure of lateral‐gate EGT: the diagram illustrates the multi‐layer and lateral structures of the single unit of the array. Multi‐layer structure of substrate/drain and source pattern as well as channel/isolation/gate pattern/electrolyte is shown. c) Notion on simulated short‐and long‐term plasticity as well as complicated synaptic function. d) Mechanism of device conductivity modulation and Li^+^ transportation.

In this study, we realized high‐density integration of EGTs to demonstrate the feasibility of the proposed integration scheme and designed the PEO/PVP/LiTFSI/ZIF‐67 electrolyte framework to enhance the array functions. Using this scheme, we integrated 100×100 EGTs on a 2.5 × 2.5 cm2 glass, as shown in **Figure** **1**a. We measured the long‐term potential and depression circles of several devices in this array and performed pattern memory and forgetting functions, with each pixel matching an individual device. Handwriting recognition was also performed, and the recognition accuracy of the representative single device was ≈92.80% with the standard deviation of the selected devices being limited to 9.69%. The realization of two image processing functions exhibited the high performance and homogeneity of this array, demonstrated the feasibility of the integration scheme, and provided a solid reference for EGT arrays with both high density and accuracy.

## 2 Results and Discussion

### 2.1 Structure Design of the Lateral‐Gate EGTs Array

A simple diagram of a logic circuit consisting of 100 × 100 lateral‐gate EGT, photos, and micrographs is shown in Figure 1a. The EGT uses a lateral gate and a thin film of electrolyte to connect the gate and channel, modulating device conductivity. However, the lateral‐gate structure applied in this study differs from the mature lateral‐gate structures utilized in the field of EGTs in that the lateral gate and source/drain (S/D) are not located in the same plane. Conversely, an isolation layer of SiO_2_ was deposited to separate the gate and S/D layers, thus preventing any connection between these layers, through which girds comprising connection lines were formed. Figure 1b shows the schematic of the multi‐layer structure of a single EGT unit utilized in the array. The drain and source electrode pattern layers were located on the TFT glass substrate. The isolation layer was then deposited to separate the drain and source from the gate‐pattern layer. After forming the pattern using gate‐pattern layer deposition and photo‐lithography, the channel region was exposed through etching to deposit the channel material. Finally, the electrolyte layer was dropped‐cast on the surface of the array. The detailed fabrication process is described in the method section. The photo‐lithography layout of the entire array and a single unit are shown in Figure S1 (Supporting Information). The read operation is carried out by applying a voltage bias on the drain grid line and the write operation is carried out by applying voltage pulses on the gate line. With both voltage bias on specific drain grid line and pulses on specific gate grid line together, the individual EGT which connected both the specific drain and gate grid line is selected, and then the conductance of its can be changed and readout.

In biological synapses, synaptic plasticity is primarily classified into short‐ and long‐term plasticity. The short‐term plasticity is relevant to intermittent stimulus‐response while the long‐term plasticity is relevant to continuous processes such as learning and forgetting. Using the design of lateral‐gate EGT artificial synapse arrays, biologic‐like short‐term and long‐term synaptic plasticity as well as complicated synaptic functions, such as handwriting recognition, learning and forgetting, which are relevant to the array, are simulated, as shown in Figure 1c. These functions are realized by the drift and relaxation of freely movable ions in the electrolyte. With the application of a pulsed voltage on the gate of the EGTs, the freely movable ions in the gate medium drift directionally and form electrical double layers at the interface between the electrolyte and channel. When the voltage is removed, the ions at the interface tend to return to its equilibrium. However, before the ions return, next pulsed voltage is applied. The ions on the way to return and more ions at the equilibrium are forced to move and thus a lager EPSC was induced due to more dense charges at the interface. The drift and relaxation of ions modulates the conductivity of the channel, thus updating the synaptic weight.^[^ ^19^, ^40^ ^]^ In this study, while Li^+^ transports primarily through hopping with local segmental motion (PEO chain) in an amorphous phase, the porous ZIF‐67 particles provide an additional interface with the PEO matrix and generate numerous conductive channels, enhancing Li^+^ transportation and thus ionic conductivity, as is shown in Figure 1d.

### 2.2 Electrolyte Design and Characterization

**Figure 2:**
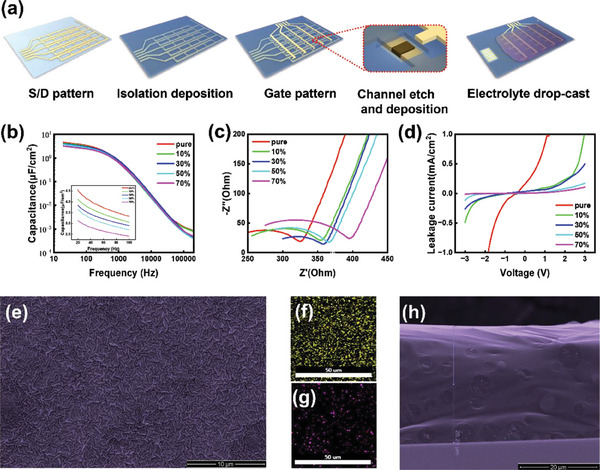
Characteristics of the electrolyte film. a) Fabrication process of the artificial synapse array. A small array consisting of 5 × 5 synaptic devices is shown. At the channel etch and deposition step, a single unit instead of the entire array is shown to illustrate the detail of this step. b) Specific capacitances from 20 Hz to 2 MHz. The specific capacitances at 20 Hz (the inserted figure) of the electrolytes with 30%, 50%, and 70% polyvinyl pyrrolidone (PVP) were 3.83, 3.65, and 3.14 µF cm^−2^, respectively. c) Electrochemical impedance characteristics. d) Leakage current of the electrolyte films with different PVP concentrations. Through trade‐off between high capacitance and low leakage, the concentration of 50% was finally confirmed to construct the synaptic device. e) Scanning electron microscopy (SEM) image of the surface of the electrolyte film. The energy‐dispersive X‐ray spectroscopy (EDS) of elements F (represented for LiTFSI) and Co (represented for ZIF‐67) are presented, as shown in f,g), respectively. h) Thickness of the electrolyte film is confirmed as 26.29 µm through the SEM image of the cross section of the electrolyte film.

As shown in **Figure** **2**a, at the final step of fabricating the high‐performance synaptic device array, an electrolyte with high ionic conductivity and a noticeable electric double‐layer effect is drop‐casted, and then solid‐state dielectric is formed. Therefore, this study characterizes the electric performance and morphology of the key functional layer. Based on results from previous reports,^[^ ^41^, ^42^ ^]^ the composition ratio of the PEO:LiTFSI:ZIF‐67 was first set to meet the requirement that the molecular ratio EO:Li^+^ is 8:1 and the mass ratio ZIF‐67:PEO is 3%. However, an electrolyte prepared with these ratios exhibits high leakage current, preventing the normal operation of the EGT synaptic device. Therefore, PVP was added to suppress the leakage current. However, although some studies have reported that the addition of PVP can increase ionic conductivity,^[^ ^43^, ^44^ ^]^ this effect was not observed in this study. Notably, the addition of PVP suppressed leakage current and ionic conductivity. Hence, to achieve a trade‐off between ionic conductivity and leakage current, the electrical characteristic of the electrolyte was characterized using different concentrations of PVP (0, 10%, 30%, 50%, and 70%, as to the weight of PEO). The characterization was presented using capacitance–frequency, AC impedance, and leakage current. The capacitance–frequency (c–f) characteristic is shown in Figure 2b. The capacitance dropped significantly to ≈104 when the frequency increased from 20 Hz to 2 MHz, exhibiting the electric double‐layer effect. The capacitance of different PVP concentrations at low frequencies dropped with an increase in PVP concentration. The smallest capacitance was ≈3.14 µF cm^−^2 when the PVP concentration was ≈70%, notably dropping from 3.65 µF cm^−^2 at 50%. When the PVP concentration was increased to 100%, the capacitance at low frequency dropped significantly, and the span of the capacitance from low to high frequency became particularly short, indicating a loss in ionic conductivity. Therefore, the characterizations of PVP concentrations exceeding 70% were not performed. Figure 2c shows the AC impedance curves of four electrolytes where the impedance curves consist of an arc caused by the charge transfer process at high frequency and a straight line with a slope caused by the mass transfer process at low frequency.^[^ ^45^, ^46^ ^]^ The horizontal ordinate of the inflection points representing the bulk resistance of the electrolyte (*R_0_*) is related to the ionic conductivity (*σ*) through the equation

(1) $$\sigma =\frac{L}{R_{0}S}$$

where *L* represents the thickness of the film electrolyte, and *S* represents the area of the electrode. A noticeable right shift of *R*_0_ occurred upon adding PVP and then grew slowly until the concentration of PVP reached 50% (366 Ω), whereas the leakage current dropped significantly to 0.17 mA at 3 V, as shown in Figure 2d. When the concentration continued to increase to 70%, *R*_0_ increased significantly. However, compared with the significant loss in ionic conductivity when the concentration increased to 70%, only a limited drop was observed in leakage current. Therefore, the concentration of PVP was finally determined as 50%. To evaluate the impact of various PVP concentration on device, the I_D_‐V_G_ curves of the device were tested, as shown in Figure S2 (Supporting Information). When PVP concentration was lower than 30%, the I_D_‐V_G_ curves did not show a transfer characteristic, indicating the loss of TFT functions caused by uncontrollable gate leakage current. When PVP concentration increased to 30%, the I_D_‐V_G_ curves began to show transfer characteristic. And with the increase of PVP concentration, the hysteresis began to shrink and the on‐off ratio decrease. A further characterization of the specific impact of PVP concentration on the synaptic performance is carried out later. Subsequently, further characterization, including the scanning electron microscope (SEM) figure of the surface and the cross section of the electrolyte, on the electrolyte containing 50% PVP was performed, as shown in Figure 2e,h. The SEM figure show that the surface exhibits relatively high homogeneity and limited roughness. This result is confirmed using energy dispersive spectrometer (EDS) on F (represented for LiTFSI, Figure 2f) and Co (represented for ZIF‐67, Figure 2g). Considering the cross section figure, the thickness of the electrolyte was ≈26.29 µm. The value of *σ* is 2.87 × 10^−3^ S cm^−1^. The high ionic conductivity of the electrolyte resulted from the high porosity and large specific surface area of ZIF‐67. Figure S3 (Supporting Information) shows the transmission electron microscopy (TEM) and high‐resolution TEM (HRTEM) figures of the ZIF‐67 particles. As shown in the HRTEM figure, the surface of ZIF‐67 was full of pores, accounting for its large specific surface area.^[^ ^47^ ^]^ To confirm the actual characteristic of the pores in the ZIF‐67 particles, a Brunauer–Emmett–Teller (BET) measurement was performed. Figure S4a (Supporting Information) shows the N_2_ adsorption/desorption curves of the ZIF‐67 particles. The isotherm shows a typical type‐I characteristic, which results from the filling of the micropores in these particles.^[^ ^48^, ^49^ ^]^ Figure S4b (Supporting Information) shows the pore distribution, indicating that the pores in the ZIF‐67 particles are primary micro‐pore. The detailed pore characteristic is shown in Table S1 (Supporting Information). Follow the mentioned design rationale, the lateral‐gate design fit similar quasi‐solid electrolyte framework with polymer matrix theoretically. To judge whether the concept of the lateral‐gate structure of EGTs can be extrapolated to other polymer‐based materials, a I_D_‐V_G_ test on device with pure PEO as electrolyte was conducted to compare with the designed electrolyte framework. The I_D_‐V_G_ curve showed a typical transfer characteristic though relevant parameters were not ideal, as shown in Figure S5 (Supporting Information). At least, it confirms that the universality of the lateral‐gate design. An investigation on the effect of electrolyte thickness on the EGTs was also carried out, as shown in Figures S6–S8 (Supporting Information). No evident deviation was observed.

### 2.3 Synaptic Functions Realized by Single Array Unit

**Figure 3:**
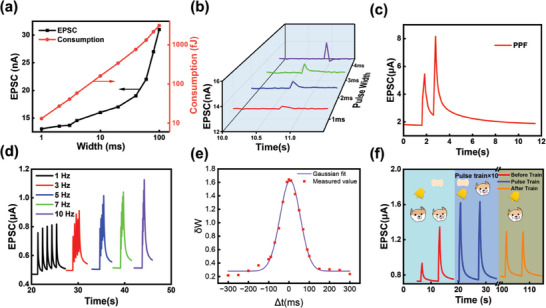
Synaptic functions realized by single unit in array. a) Power consumption at different pulse widths from 1 to 100 ms. b) EPSC response at extremely short width from 1 to 4 ms. c) Pair‐pulse facilitation of a representative unit in array. d)Frequency‐dependent EPSC response stimulated by five continuous pulses. e)Spike‐timing dependent plasticity. f) Pavlovian experiment simulation.

Basic synaptic functions such as excitatory postsynaptic currents (EPSCs) at different pulse amplitudes, widths, and amounts (Figure S9, Supporting Information) were simulated using a single EGT in the array. Power consumption was calculated according to various pulse widths, as shown in **Figure** **3**a. Notably, a single EGT could operate at an extremely low consumption of 27.06 fJ per synaptic event because it could provide evident EPSC response at an extremely small drain‐source bias of 1 mV and extremely short pulse width of 2 ms, as shown in Figure 3b. A comparison based on device amounts and energy consumption of one synaptic event of single device was carried, as shown in Figure S10 (Supporting Information), indicating competitive energy consumption performance for further application.

Figure 3c shows the pair‐pulse facilitation (PPF) behavior simulated by the synaptic device. PPF is an important neuron behavior consisting of two continuous EPSCs caused by two pulses, where the latter EPSC is larger than the former one, indicating the synaptic weight update.^[^ ^11^, ^12^ ^]^ PPF behavior is a fundamental synaptic function accounting for short‐term memory in biological neuron. Scholars use PPF index to describe PPF ability quantificationally:

(2) $$PPF=\frac{A_{2}}{A_{1}}$$

where *A_1_* represents the amplitude of the former EPSC while *A_2_* represents the amplitude of the latter one. A representative PPF index of the single unit in this array at the pulse interval Δ*t*  =  1*s* was 1.73. The PPF index of this work is competitive and favorable for the simulation of short‐term synaptic function.

In biological neuron, neurotransmitter is sent by vesicles, which are selectively responded by post‐synaptic terminal depending on vesicles releasing frequency, similar to the high‐pass filtering in conventional computer.^[^ ^50^, ^51^ ^]^ Several series of five pulse signals with different frequency were applied on the gate to simulate high‐pass filtering, and it is observed in Figure 3d that the higher frequency was, the larger EPSC became. The relative variation could be taken as a criterion of frequency to judge whether the input signal is of high enough frequency since that higher frequency results for larger relative variation, which is illustrated in Figure S11 (Supporting Information).

Spike‐timing dependent plasticity (STDP) shows the synaptic plasticity depending on the sequence of the pre‐synaptic potential (pulsed V_G_), the post‐synaptic potential (pulsed V_D_) and the interval, which are considered as the base of learning.^[^ ^52^, ^53^ ^]^ In this work, STDP characteristic was shown through the synapse weight relative variation ΔW as a function of the interval Δt between the pre‐synaptic potential and the post‐synaptic potential. The symbol of Δt was positive when the pre‐synaptic potential came later than the post‐synaptic potential and negative when the pre‐synaptic potential came earlier than the post‐synaptic potential. STDP function was successfully realized and the measured result was shown in Figure 3e. The STDP behavior was highly symmetric and its distribution was accordant with Gaussian function as the fitting line showed. ΔW dropped dramatically from 1.6 to 0.5 when the absolute value of Δt approached 100 ms and then remained almost the same when Δt kept increasing.

Pavlovian experiment introduced the concept of conditioned reflex, which connect the learning and memory.^[^ ^54^, ^55^ ^]^ In this work, pulsed V_G_ with amplitude of 1 and 2 V were utilized to simulate the bell ring and the food, respectively. It was observed in Figure 3f that the EPSC caused by the pulsed V_G_ of 1 V was within 1 µA while the EPSC caused by the pulsed V_G_ of 2 V exceeded 1 µA. Taking 1 µA as the baseline, EPSC exceeded 1 µA represented that the dog slavered. Then a series of combined pulsed V_G_ of 1 and 2 V were applied to implement the learning process. After ten combined pulses, the EPSC caused by the pulsed V_G_ of 1 V also exceeded 1 µA, indicating the memory. The successful simulation of Pavlovian experiment showed the high performance of the synaptic device in long‐term plasticity, which is favorable for the further investigation.

### 2.4 Long‐Term Plasticity of the Artificial Synapse Array

An artificial synapse array with high performance and homogeneity is required to ensure the applicability of the synaptic device. Except for the aforementioned synaptic function, long‐term potential and depression circles are required to test synaptic weight control. Precise synaptic weight control is necessary to construct hardware neural networks as it can determine the pulses required to change the synaptic weight to a certain value. In a single circle, as the EPSC caused by one pulse tends to be saturated when the pulse number increases, the same synaptic weight variation requires fewer pulses at the beginning of the potential (depression) than at the end, indicating a linear change in synaptic weight in a single potential (depression) process. In contrast, in multiple circles, more or fewer pulses may be required to achieve a certain synaptic weight, indicating the stability of the synaptic device.^[^ ^56^ ^]^ Hence, to characterize the linearity and stability of the synaptic device, we used two parameters termed nonlinearity (NLP and NLD for long‐term potential and depression, respectively) and circle‐to‐circle (CTC) variation expressed by the equation:

(3) $$G_{p}=B_{p}\left(1-e^{\frac{-P}{A_{p}}}\right)+G_{min}$$

(4) $$G_{d}=-B_{d}\left(1-e^{\frac{P-P_{max}}{A_{d}}}\right)+G_{max}$$

(5) $$B_{p,d}=\frac{G_{max}-G_{min}}{1-e^{\frac{-P_{max}}{A_{p,d}}}}$$

(6) $$CTC=\frac{\sqrt{\frac{\sum_{j}^{m}\sum_{i}^{n}{\left(G_{i,j}-G_{i,average}\right)}^{2}}{mn}}}{G_{max}-G_{min}}$$

where *G* represents the conductivity of the channel, which acts as the synaptic weight mentioned before; subscripts *p* and *d* represent the long‐term potential and long‐term depression, respectively; *A* is a parameter related to nonlinearity; *P* represents the pulse number; *i* represents the *i*_th_ pulse in one circle; *j* represents the *j*_th_ circle of one synaptic device; *m* and *n* represent the total circle number and the total pulse number, respectively; *G*_i,average_ represents the average conductivity of the *i*_th_ pulse number in the measured circle; *G*_max_ and *G*_min_ are the maximum and minimum conductivities, respectively. In this study, because of the lack of a peripheral circuit and the large scale of the array, 225 out of 10000 synaptic devices in the array were selected to run the potential and depression circles. To confirm the representativeness, the 225 synaptic devices were distributed evenly in nine areas of the array and each area contained 25 synaptic devices. Each synaptic device performed three successive potential and depression circles (each consisted of 60 potential and 60 depression states) and relevant parameters were recorded. Relevant measurement parameters and data processing methods are described in Supporting Information. Also, the long‐term potential performances of various PVP concentrations (30%, 50%, 70%) were tested, as shown in Figure S12 (Supporting Information). Although devices with PVP concentrations of 30% show increasing conductance in this process, the EPSCs waves fluctuate dramatically with irregular EPSCs peaks and troughs as a result of big current leakage. Although fluctuation with abnormally large EPSCs peaks is not observed in device with PVP concentration of 70%, the EPSCs saturate easily due to the relatively poor ionic conductivity.

**Figure 4:**
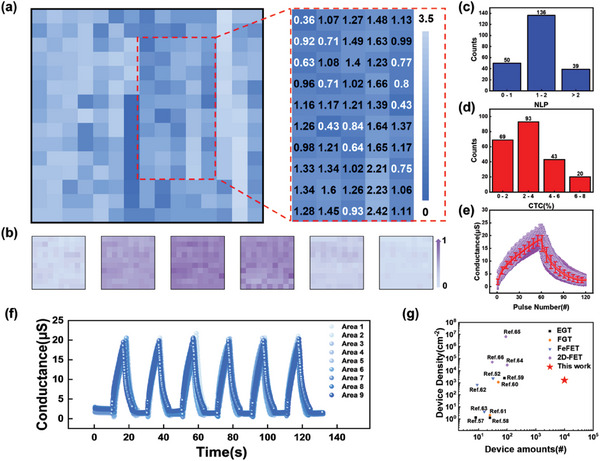
Long‐term plasticity measurement of 225 synaptic devices selected from the array. a) NLP mapping and enlarged view with specific NLP values. b) Mapping of synaptic weight updated process during potential and depression. c,d) Bar charts of the statistical results of the NLP and circle‐to‐circle (CTC). e) The second potential and depression circle of all devices evenly distributed in nine areas. Average fitting line with error bars is also provided. f) Six circles ran by nine representative devices evenly distributed in this array. g) Previous works on integration of transistor‐based synaptic device. The types of synaptic devices include EGT,^[^ ^57^, ^58^, ^59^ ^]^ floating‐gated transistor (FGT),^[^ ^60^, ^61^ ^]^ ferroelectric field‐effect transistor (FeFET),^[^ ^53^, ^62^, ^63^ ^]^ and transistor with 2D materials as channel (2D‐FET).^[^ ^64^, ^65^, ^66^ ^]^

The NLP of all the tested devices and an enlarged view with specific NLP values are shown in **Figure** **4**a. Blue sharing similar depth is shown in the map, indicating the homogeneity and relative supreme performance of the synaptic device array. Figure 4b shows the normalized updated synaptic weight of a part of the array during the potential and depression processes. With the same pulse amounts, the synaptic weights of the individual devices exhibited similar changes, indicating similar control on the synaptic weight shared by all the devices in this array. After statistical treatment, bar charts on the NLP and potential nonlinearity of all the synaptic devices are shown in Figure 4c,d. The average NLP of these devices was 1.44, and the average CTC was limited to 3.10%. Based on the statistical result, most devices exhibited high performance, including a small CTC and relatively small nonlinearity. Figure 4e depicts the second circles run by all the devices, where the average (of all the 225 devices) fitting line is represented by an error bar. No noticeable difference was observed in the performance of all the synaptic devices. Nine representative devices were evenly selected from nine areas to run six long‐term potential and depression circles, as shown in Figure 4f. The almost identical and overlapped curves depicted in each circle indicate the excellent stability of these synaptic devices along with the high homogeneity. To summarize, the integrated devices in this array exhibited low nonlinearity, high uniformity, and high stability, which is crucial for neuromorphic computation. Lastly, the integrated array reported in this study presents excellent density and scale when compared to those reported in previous studies. Figure 4g presents several previous works varying from the EGTs and other transistor‐based synaptic devices. The density and amounts of integrated devices presented in this study provide a solid reference for the integration of transistor‐based synaptic devices.

### 2.5 Image Processing Functions Realized by the Artificial Synapse Array

**Figure 5:**
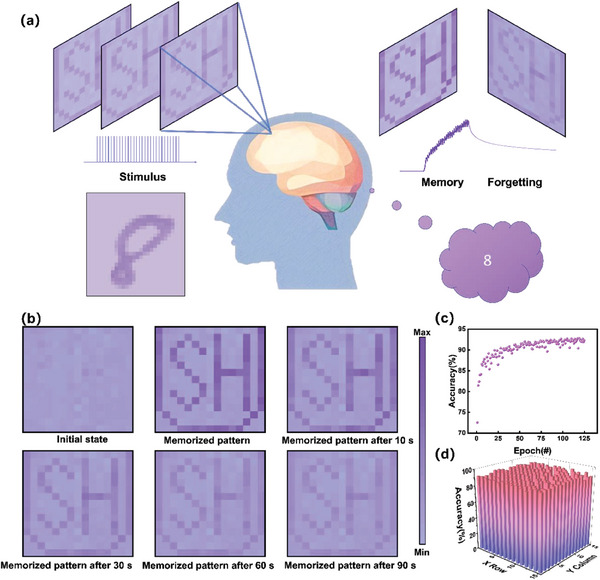
Image processing functions based on artificial synapse array. a) Diagram of brain processing pattern memory and forgetting, and handwriting recognition. b) Pattern memory and forgetting processes. Pattern is recorded at the beginning and end of the memory process, 10, 30, 60, and 90 s after memory. c) Typical training process of the recognition accuracy. d) Distribution bar graph of recognition accuracy.

Image processing functions were simulated based on the link between pixels and individual devices to evaluate the performance of the array for the application of neuromorphic computation and to emphasize the superiority of the large array. As a basic image processing function, pattern memory and forgetting processes were performed, which reflect the array homogeneity of a long‐term potential and corresponding retention time, as shown in **Figure** **5**a. The aforementioned 225 devices distributed evenly in the array were divided into pattern and background parts. The devices in pattern part were stimulated by 30 pulses of *V_G_* = 3 V, *W* = 50 ms, and *f* = 10 Hz to form a pattern. The resulting EPSC was then recorded to simulate the pattern memory process. The EPSCs of these devices were recorded after 10, 30, 60, and 90 s, to simulate the forgetting process. All the EPSCs measured at each synaptic device and state were normalized to determine the relative deviation among the 225 devices, including the pattern and background parts. Notably, every pixel of the image was made though stimulus on an individual device in the array. As shown in Figure 5b, the initial states of the 225 devices are almost the same. When the pulsed voltage was applied on specific synaptic devices, the pattern emerged and the color of the pattern deepened, indicating the memory process. Notably, the colors of the stimulated pixels were almost the same and formed noticeable distinctions from the background, confirming the homogeneity of the EPSC after the same stimulation. When the pulsed voltage was removed, the EPSC decreased but remained in a larger state than the initial state, which was reflected as a fading but still distinct pattern. After 90 s, the pattern was still noticeable and distinct from the background, indicating the long retention time of the synaptic devices. The pattern memory and forgetting process simulation resulted from the supreme performance and high homogeneity of the artificial synapse array.

After pre‐processing potential and depression data, a handwriting recognition computation according to the Modified National Institute of Standard and Technology (MNIST) database was performed using the framework of MLP simulator (+NeuroSim) V3.0 provided by the research group of Prof. Shimeng Yu.^[^ ^67^ ^]^ The artificial neuron network used in this simulation is a typical multi‐layer perceptron. It consisted of an input r, a hidden, and an output layer, including 784 (28 × 28) input, 400 hidden, and 10 output neurons, respectively. The scale of the input neurons (784) was determined by an image scale of 28 × 28 pixels provided by MNIST. When the pixel information, which is the grayscale value, was input into the input layer, synapses between the input and hidden neurons processed and transferred the data. The processing, which was primarily a simple multiplication, used the synaptic weight of the synaptic device, where the aforementioned precise control is relevant. Following the weighted summation of all the data, the 10 output neurons output 10 values, where the label of the neuron that outputs the biggest value is the number recognized by the artificial neuron network. If the label corresponds to the input number, the weights of all the synapses are recorded and used for the next training. Otherwise, their weights are changed to a suitable value, rendering the final number recognized consistent with the input number. The selected 225 synaptic devices all performed the handwriting recognition computation to test the performance and homogeneity of the array. A representative training process is shown in Figure 5c. The recognition accuracy was up to 92.8% at epoch #119. A distribution chart of the recognition accuracy of the 225 synaptic devices is shown in Figure 5d. The average and median accuracies of the recognition were 88.01% and 91.60%, respectively, whereas the standard deviation was 9.69%, validating the homogeneity of the array. Using the training process shown in Figure 5c, an intuitional training process simulation depending on a specific graph is performed, as shown in Figure S13 (Supporting Information). With an increase in accuracy, the image of the number “8” became significantly distinct owing to the weighted summation of the pixel values.

## 3 Conclusion

In this study, a high‐density EGT‐based synaptic device array was designed to verify the feasibility of the integration scheme of EGTs based on the lateral structure using conventional inorganic semiconductor technologies, particularly photo‐lithography. Using this scheme, 100 × 100 EGTs were integrated into a glass of 2.5 × 2.5 cm^2^, with a density of ≈1600 devices cm^−^2. To test the performance of the array, an electrolyte framework of PEO/PVP/LiTFSI/ZIF‐67 with high ionic conductivity of ≈2.87 × 10^−3^ S cm^−1^ benefitting from high porous ZIF‐67 particles was designed. The electrolyte exhibited a high specific capacitance of ≈3.65 µF cm^−^2 and a low R_0_ of 366 Ω. Limited roughness and high homogeneity were observed using SEM and EDS. With the superior electrolyte framework, the array successfully realized synaptic functions including EPSC, pair‐pulse facilitation, high‐pass filtering, spike‐timing dependent plasticity, and Pavlovian experiment simulation. Notably, the synaptic device in the array could respond to extremely short signals of 2 ms under an extremely small bias of 1 mV and therefore operated with an extremely small energy consumption of 27.06 fJ per synapse event. Long‐term potential and depression circles of evenly selected 225 devices were tested to extract the synaptic characteristic of the synaptic devices. A pattern memory and forgetting process was also simulated, with every pixel of the image being matched with an individual device, and a noticeable distinction between the memorized pattern and background was observed even after 90 s. Handwriting recognition was also performed, and the recognition accuracy of the representative single device was ≈92.80% and the standard deviation of all the devices was limited to 9.69%. The high‐density integration of the EGTs with high performance and homogeneity verified the feasibility of the scheme based on lateral‐structure EGTs and provided a solid reference for the integration of EGT synaptic devices. However, the synaptic device array requires additional peripheral circuit to update the synaptic weight of each synaptic device and relevant algorithm to make the update process reasonable. Although difficult, the process is promising where more efforts from each relevant subject are necessary.

## 4 Experimental Section

### Fabrication of Array Except for Electrolyte Layer

A piece of thin‐film transistor glass substrate was cleaned with acetone, ethanol, and deionized water using an ultrasonic cleaning device and then dried in N_2_ atmosphere. Using magnetron sputtering on the glass substrate, 150 nm Mo and 50 nm indium tin oxide (ITO) drain (source) electrode layers were deposited. The pattern of the electrode layer was then formed through photo‐lithography depending on the layout. A 300‐nm‐thick SiO_2_ isolation layer was deposited via plasma‐enhanced chemical vapor deposition to separate the drain (source) electrode and gate electrode layers. Subsequently, the gate electrode layer was fabricated in the same process as the drain (source) electrode layer. Dry etching was then performed on the isolation to expose the channel regions, and InGaZnO (IGZO) with a thickness of 150 nm was deposited via magnetron sputtering. To ensure close contact between the IGZO channels and drain (source) electrodes, photo‐lithography was performed to align the channels to the electrodes, and the residual IGZO was dry etched. The array with the IGZO channels was then annealed at 220 °C for 2 h to reduce defects in the sputtered IGZO and enhance conductive performance. At this moment, the EGT array without the electrolyte layer was fabricated.

### Preparation of the Composite Electrolyte

ZIF‐67 was milled, dried, and then activated at 110 °C in a vacuum to eliminate moisture in its porous structure. The activated ZIF‐67, PEO with an average Mv of 600000, LiTFSI, and PVP with an average Mv of 130000 were transferred into a glass bottle. The ratio satisfies the following requirement: the molecular ratio EO:Li^+^ = 8:1, the mass ratio ZIF‐67:PEO = 3%. Different PVP concentrations meet the mass ratio PVP:PEO = 0, 10%, 30%, 50%, and 70%. Acetonitrile was then added in the ratio 1 g PEO:24 ml acetonitrile. The turbid liquid containing the PEO/PVP/LiTFSI gel and ZIF‐67 particles was stirred for 12 h to achieve maximum homogeneity. After stirring, 400 ml of the electrolyte gel was transferred to the array through drop‐casting and dried in vacuum.

### Methods of Characterizing the Electrical Performance of the Electrolyte

After stirring, 400 ml of the electrolyte gel was transferred to a patterned ITO glass through drop‐casting, which was then dried in vacuum. After drying, a patterned aluminum layer was deposited through vacuum thermal evaporation to form a sandwich structure of an ITO electrode/electrolyte/aluminum electrode. The measurements of the electrical performance of the electrolyte including the capacitance–frequency, AC impedance, and leakage were performed using an impedance analyzer (Agilent E4980A) and electrochemical workstation (CHI760E). The frequency span of the measurement was from 20 Hz to 2 MHz with a scanning voltage of 5 mV.

### Electrical and Synaptic Function Measurement

The synaptic function was measured using Keithley 4200A‐SCS. The pulsed signal was input using a pulse generator (RIGOL DG4062). Detailed measurement parameters are described in the Result Section and Supporting Information.

## Conflict of Interest

The authors declare no conflict of interest.

## Author Contributions

J.L. and J.Z. directed this work and supervised the process and result. Y.L., Z.W., and S.W. performed the array test. H.M., Q.T., Z.L., and W.G. fabricate the array. Y.L. obtained data and wrote the manuscript, and revised it under guide of J.L. and M.L., and W.Z. simulated handwriting recognition.

## Supporting information

Supporting Information

## Data Availability

The data that support the findings of this study are available from the corresponding author upon reasonable request.
