# Supplementary material for: High‐Density Artificial Synapse Array Consisting of Homogeneous Electrolyte‐Gated Transistors
Source: Adv Sci (Weinh). 2023 Nov 28;11(3):2305430. doi: 10.1002/advs.202305430 (PMC10797465; doi:10.1002/advs.202305430)
Supplement: Supplementary file 1 — Supporting Information [file ADVS-11-2305430-s001.pdf]

ADVANCED SCIENCE
Open Access

Supporting Information

for *Adv. Sci.*, DOI 10.1002/advs.202305430

High-Density Artificial Synapse Array Consisting of Homogeneous Electrolyte-Gated Transistors

Jun Li, Yuxing Lei, Zexin Wang, Hu Meng, Wenkui Zhang, Mengjiao Li, Qiuyun Tan, Zeyuan Li, Wei Guo, Shengkai Wen and Jianhua Zhang**

Supporting Information

High-density artificial synapse array consisting of homogeneous electrolyte-gated transistors

Jun Li,[a,b,c*] Yuxing Lei,[a] Zexin Wang,[a] Hu Meng,[d] Wenkui Zhang,[c] Mengjiao Li,[c] Qiuyun Tan,[d] Zeyuan Li,[d] Wei Guo,[d] Shengkai Wen,[a] and Jianhua Zhang[b,c*]

[a] School of Material Science and Engineering, Shanghai University, Jiading, Shanghai 201800, People's Republic of China.

[b] Key Laboratory of Advanced Display and System Applications, Ministry of Education, Shanghai University, Shanghai 200072, People's Republic of China.

c School of Microelectronics, Shanghai University, Jiading, Shanghai 201800, People's Republic of China

d Central Research Institute, BOE Technology Group Company, Ltd., Beijing, People's Republic of China

Measurement of the long-term potential and depression circles and relevant data process

A single long-term potential and long-term depression circle was obtained where successive 60 pulsed V_G of 3 V, 10 Hz, and 50 ms were applied on the gate and then successive 60 pulsed V_G of −3 V, 10 Hz, and 50 ms were applied. Multiple circles were obtained by repeating the same process. Then, the row data of several circles were extracted and all troughs of each circle were found and recorded as the conductance states of the synapse transistor. The conductance states and matched pulse numbers were normalized and then fitted using the equation mentioned in the main text. Then, the normalized $A_{p,d}$ was matched with the nonlinearity table provided by Prof. Shimeng Yu. Subsequently, the CTCs of the multiple circles were calculated.[1]

* Corresponding authors: lijun_yt@shu.edu.cn (J. Li), jhzhang@oa.shu.edu.cn (J.H. Zhang)

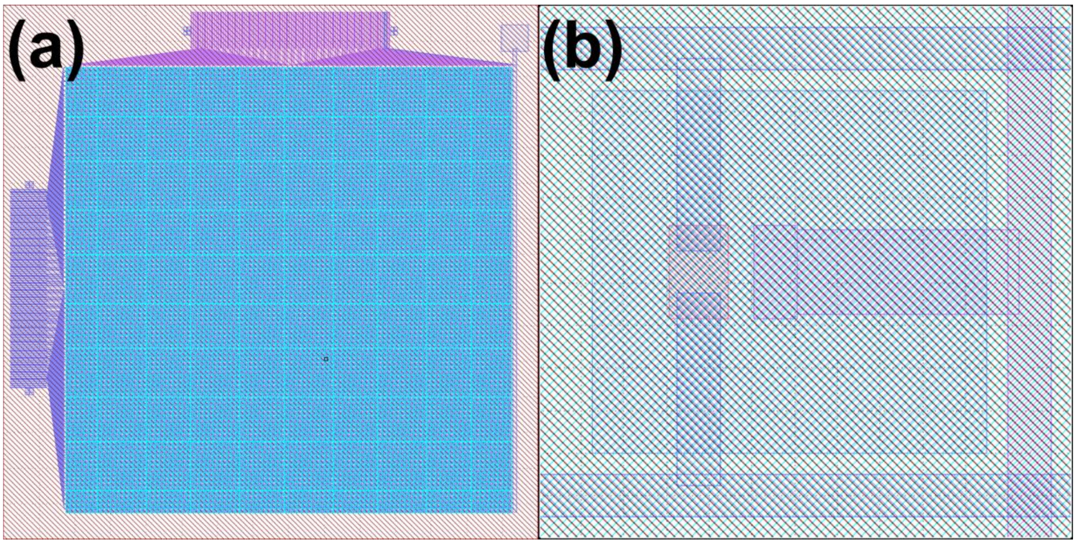

Figure S1 Photo-lithography layout of the entire array (a) and a single unit (b). In the single unit, the vertical grid lines are the gate electrodes, and the horizontal grid lines are the drain and source electrodes. These parallel and perpendicular electrode grid lines connect devices located at different rows and columns and simultaneously send pulse signals to realize the writing and reading actions. Three electrode lines are expanded from the connection grid lines, which are the horizontal gate, relevantly thinner vertical drain, and source of a single synapse transistor. Between the drain and source is a channel with a wide-length ratio of 20 μm:20 μm. The gate is aligned centrally with the channel, and its edge maintains a distance from channel of 12 μm.

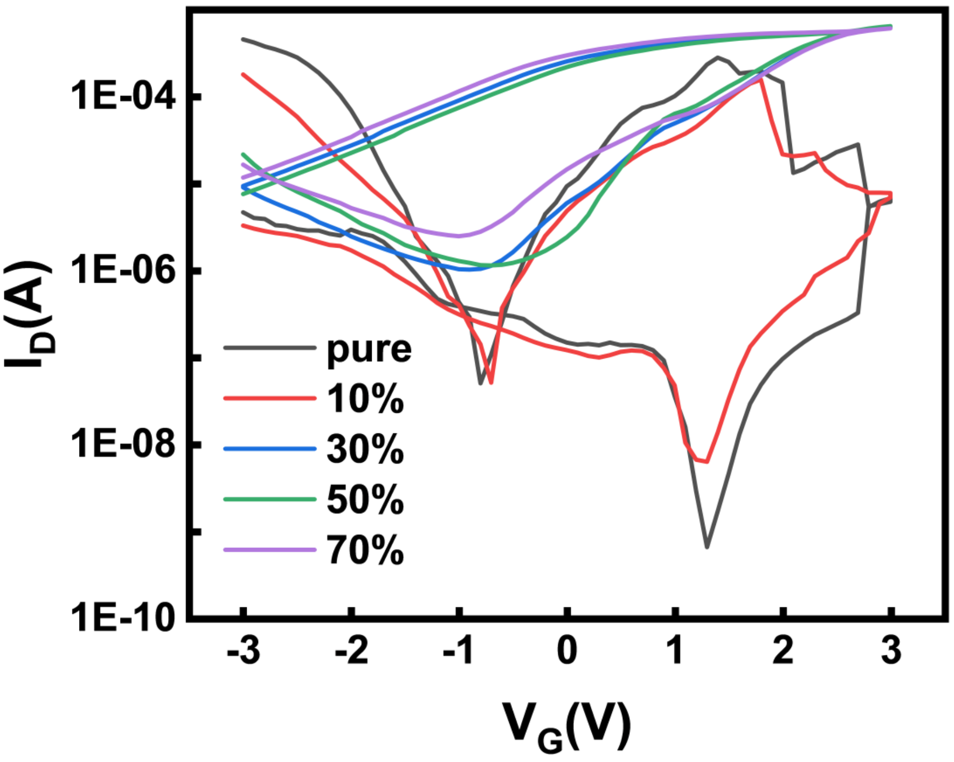

Figure S2 I_D-V_G curves of devices with various PVP concentration.

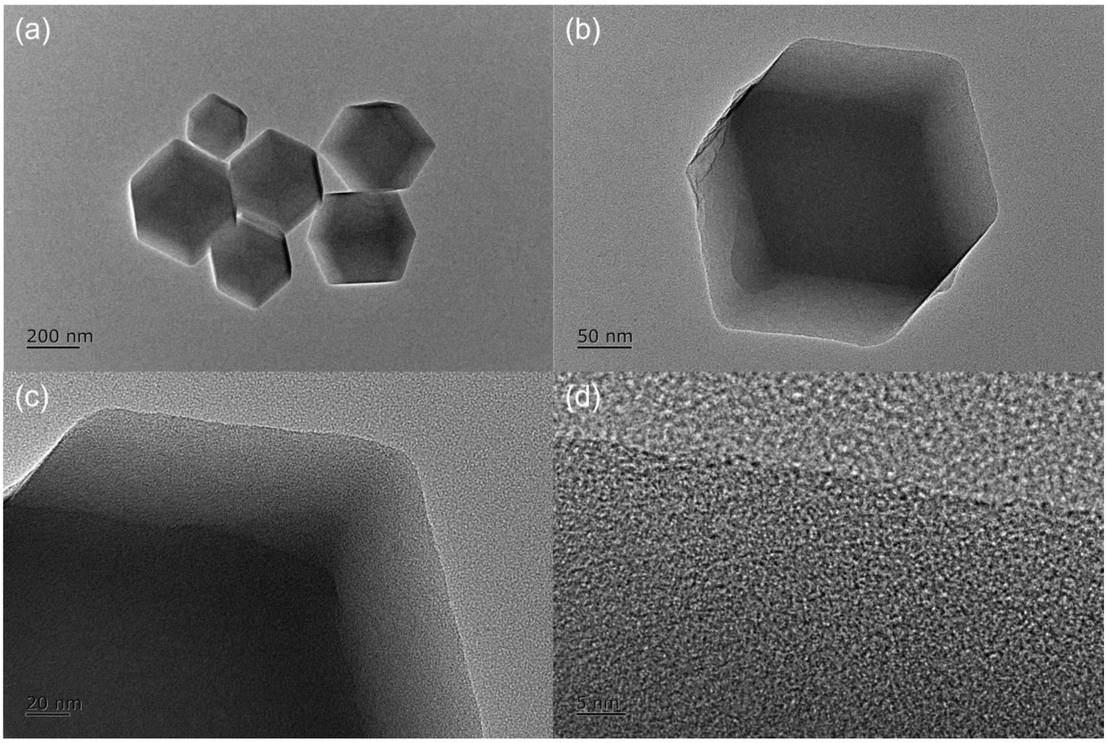

Figure S3 TEM(a)(b) and HRTEM (c)(d)image of the ZIF-67 particles

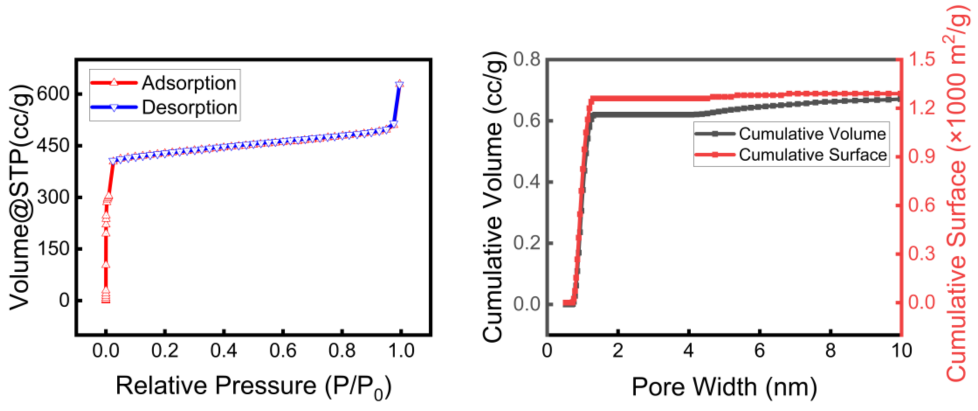

Figure S4 The BET characterization on the ZIF-67 particles. (a) Absorption and desorption isotherm. (b) Pore distribution was performed using DFT methods.

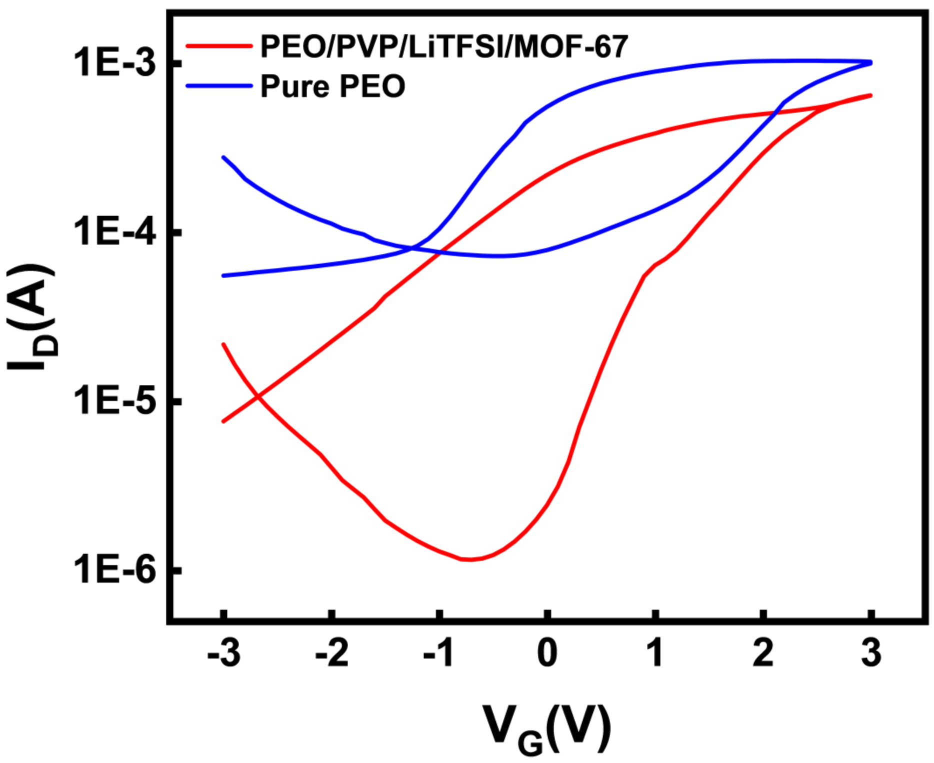

Figure S5 I_D-V_G test on device with pure PEO and PEO/PVP/LiTFSI/MOF-67 as electrolyte. The I_D-V_G curve of device with pure PEO showed a typical transfer characteristic though relevant parameters such as on-off ratio and the hysteresis were not ideal due to the poor ionic conductivity of the PEO matrix.

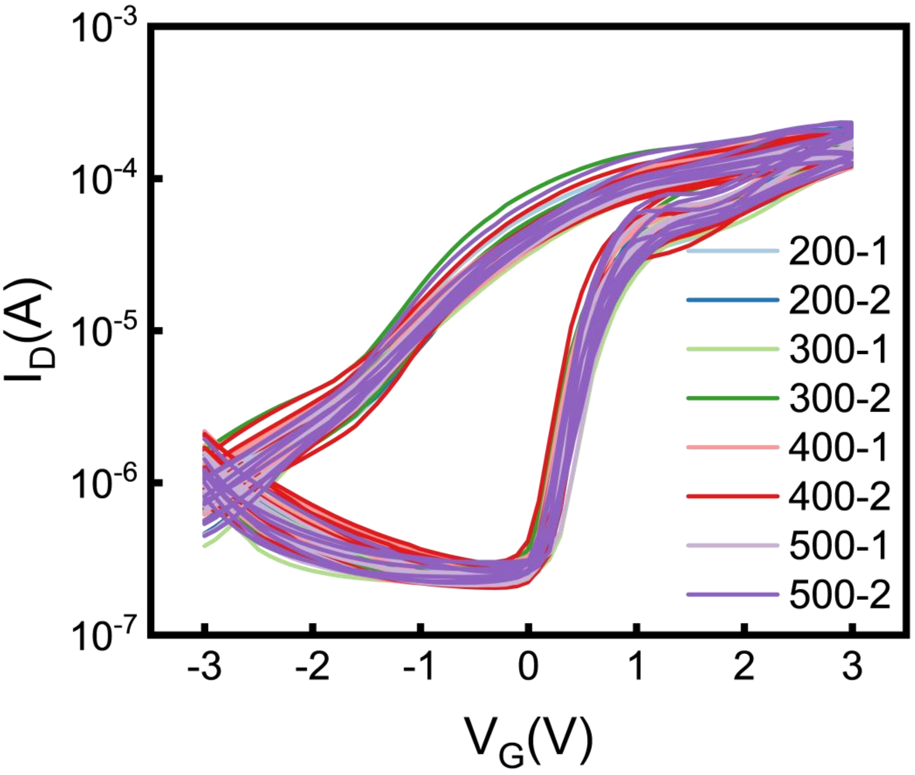

Figure S6 Transfer curves of devices in two batches with various electrolyte thickness. The thickness of electrolyte was controlled through drop-cast specific amount (200 μL, 300 μL, 400 μL, 500 μL) of electrolyte on the EGTs area(of 2.5 cm * 2.5 cm). A series of transfer curves of devices(including two batches, each batch contained eight EGTs) with various electrolyte thickness(200 μL, 300 μL, 400 μL and 500 μL) were shown. The thickness and batch were noted in legend as ”thickness-batch”. The transfer curves showed similar characteristics(including subthreshold swing of 0.266 V/dec with standard deviation of 0.021 V/dec and threshold voltage of 0.030 V with standard deviation of 0.030 V. No evident deviation was observed.

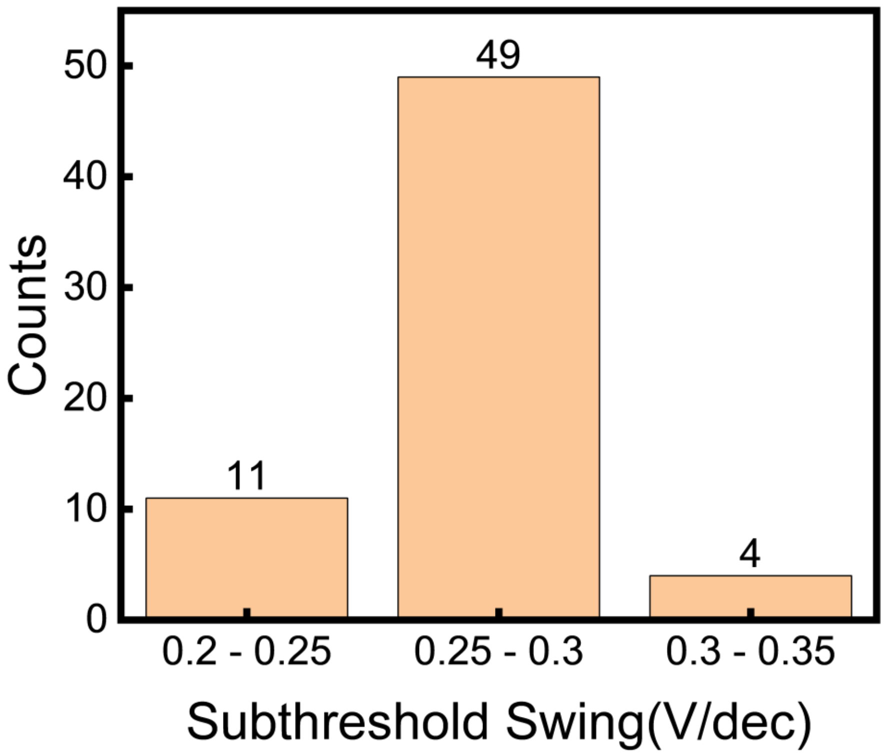

Figure S7 Subthreshold swing distribution of devices in two batches with various electrolyte thickness. The mean subthreshold swing is 0.266 V/dec with standard deviation of 0.021 V/dec.

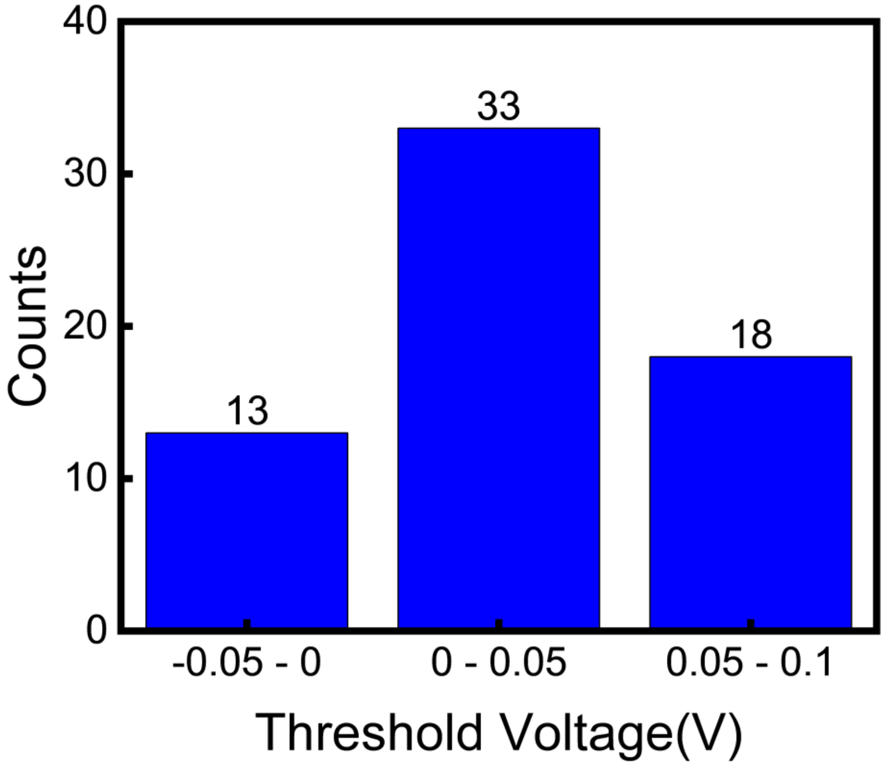

Figure S8 Threshold voltage distribution of devices in two batches with various electrolyte thickness. The mean threshold voltage of 0.030 V with standard deviation of 0.030 V.

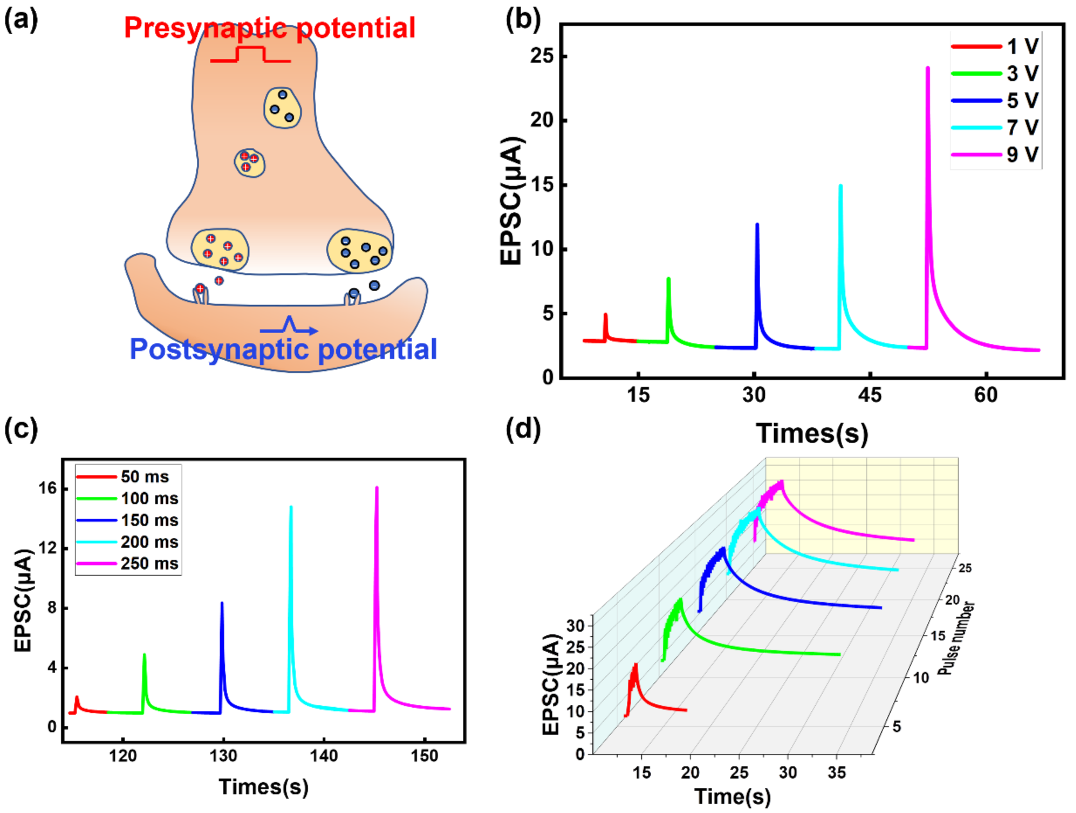

Figure S9 Basic EPSC characteristic. (a)Schematic of biological synapse. EPSC stimulated with different pulse amplitudes (b), amounts (c), and width (d). Except for the specific parameter, the base parameter has an amplitude of +3 V and a pulse width of 150 ms.

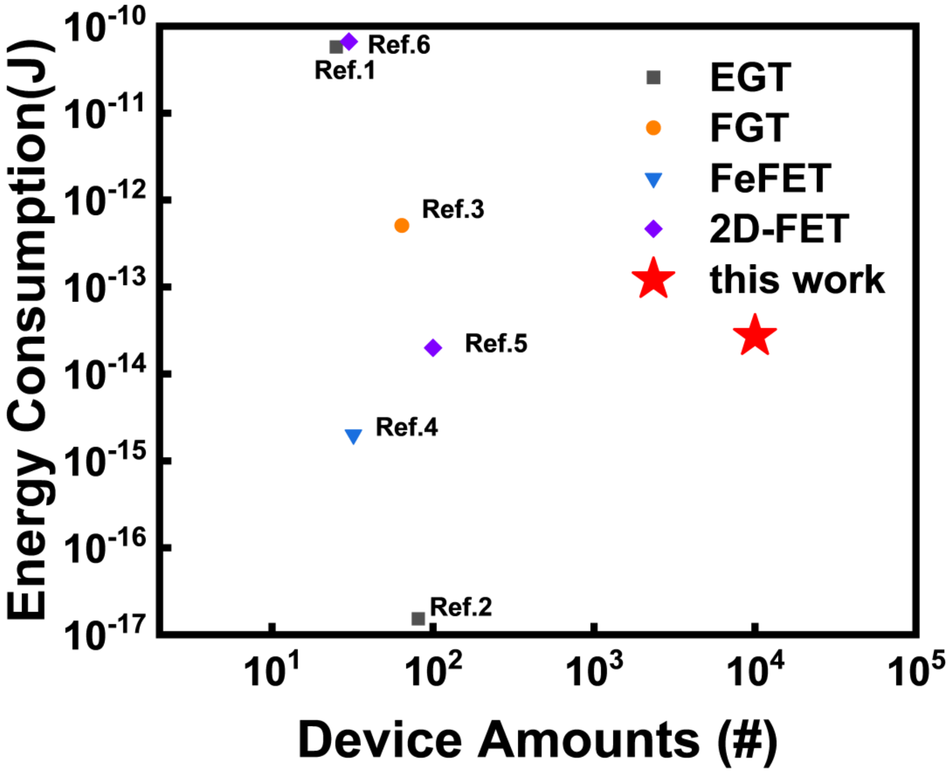

Figure.S10 Energy Consumption Comparison[1-6]**.**

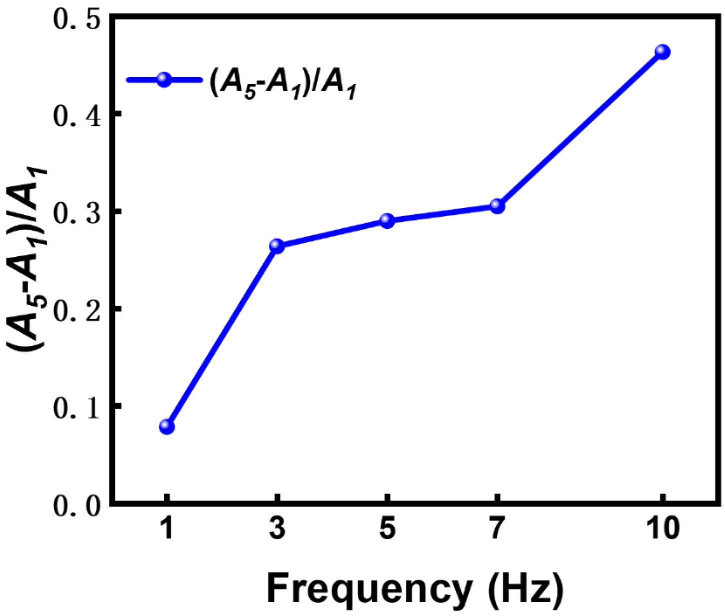

Figure S11 Relative variation of EPSCs caused by pulses with different frequency. Curve of $(A_5-A_1)/A_1$, where A_1 and A_5 represent the amplitude of the first and last induced EPSC, respectively. Frequency increases with increasing EPSC. The relative

variation could be considered a criterion of frequency as the last EPSC increases with increasing frequency. High-pass filter can be obtained by setting a threshold of relative variation and determining whether the input signal is of high enough frequency.

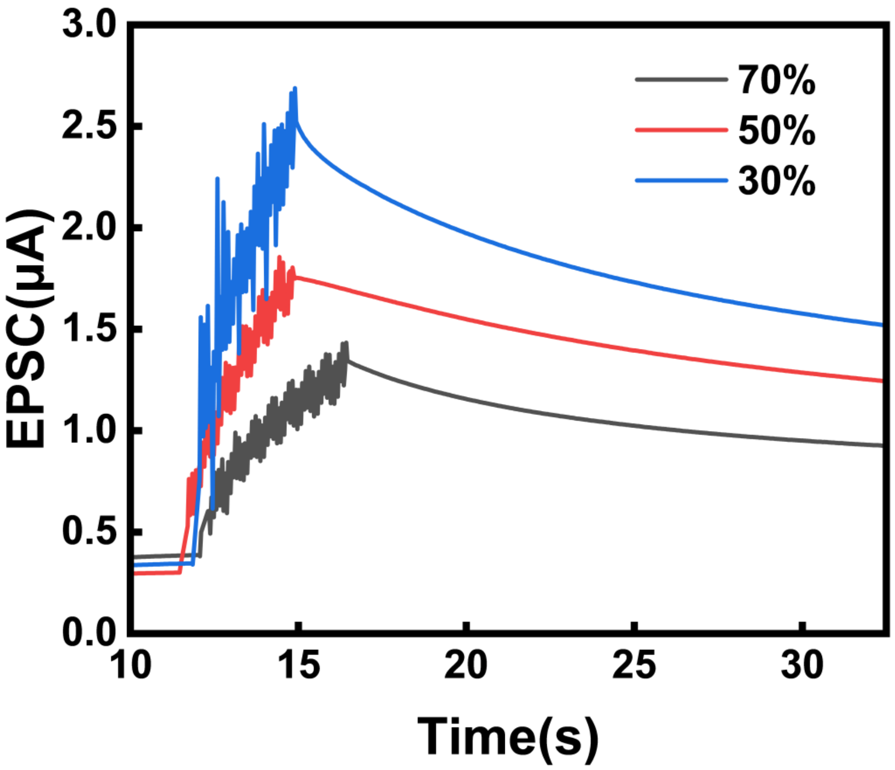

Figure S12 Long-term potential process mimicked by device with various PVP concentration. The stimulus are pulses with amplitude of 3 V, width of 50 ms, frequency of 10 Hz.

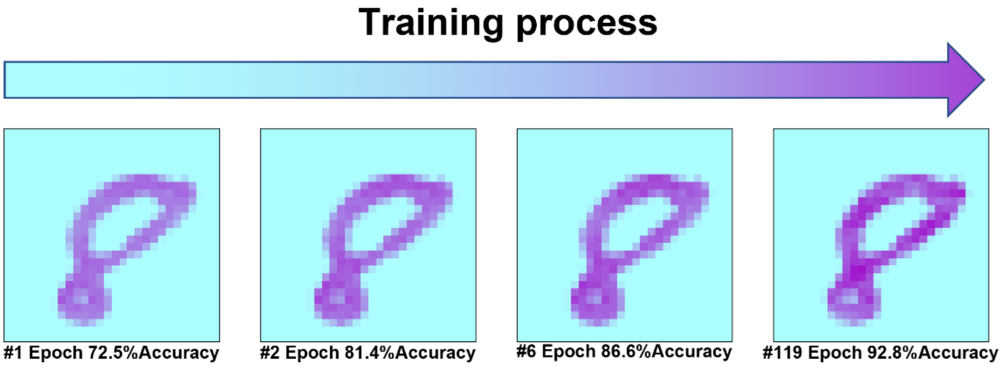

Figure S13 Typical training process simulation.

Table.S1 Pore characteristic of the ZIF-67 particles

**Pore Volume**	**0.723 cc/g**
**Surface Area**	**1303.433 m^2/g**
**Pore Width**	**0.926 nm**

Reference:

[1] C. Jin, W. Liu, Y. Huang, Y. Xu, Y. Nie, G. Zhang, P. He, J. Sun, J. Yang, *Appl. Phys. Lett.* **2022**, 120.

[2] T. Xie, Q. Wang, M. Li, Y. Fang, G. Li, S. Shao, W. Yu, S. Wang, W. Gu, C. Zhao, M. Tang, J. Zhao, *Adv. Funct. Mater.* **2023**.

[3] C. Jo, J. Kim, J. Y. Kwak, S. M. Kwon, J. B. Park, J. Kim, G. S. Park, M. G. Kim, Y. H. Kim, S. K. Park, *Adv. Mater.* **2022**, 34, e2108979.

[4] M. K. Kim, I. J. Kim, J. S. Lee, *Sci. Adv.* **2022**, 8, eabm8537.

[5] X. Feng, S. Li, S. L. Wong, S. Tong, L. Chen, P. Zhang, L. Wang, X. Fong, D. Chi, K. W. Ang, *ACS Nano* **2021**, 15, 1764.

[6] H. S. Lee, V. K. Sangwan, W. A. G. Rojas, H. Bergeron, H. Y. Jeong, J. Yuan, K. Su, M. C. Hersam, *Adv. Funct. Mater.* **2020**, 30.
